# Discriminative Shape Feature Pooling in Deep Neural Networks

**DOI:** 10.3390/jimaging8050118

**Published:** 2022-04-20

**Authors:** Gang Hu, Chahna Dixit, Guanqiu Qi

**Affiliations:** 1 Computer Information Systems Department, State University of New York at Buffalo State, Buffalo, NY 14222, USA; hug@buffalostate.edu; 2 Faculty of Computer Science, Dalhousie University, Halifax, NS B3H 4R2, Canada; chahna.dixit@dal.ca

**Keywords:** pooling, shape feature, classification, edge

## Abstract

Although deep learning approaches are able to generate generic image features from massive labeled data, discriminative handcrafted features still have advantages in providing explicit domain knowledge and reflecting intuitive visual understanding. Much of the existing research focuses on integrating both handcrafted features and deep networks to leverage the benefits. However, the issues of parameter quality have not been effectively solved in existing applications of handcrafted features in deep networks. In this research, we propose a method that enriches deep network features by utilizing the injected discriminative shape features (generic edge tokens and curve partitioning points) to adjust the network’s internal parameter update process. Thus, the modified neural networks are trained under the guidance of specific domain knowledge, and they are able to generate image representations that incorporate the benefits from both handcrafted and deep learned features. The comparative experiments were performed on several benchmark datasets. The experimental results confirmed our method works well on both large and small training datasets. Additionally, compared with existing models using either handcrafted features or deep network representations, our method not only improves the corresponding performance, but also reduces the computational costs.

## 1. Introduction

Computer vision aims to enable computers to emulate the human visual system by learning and making inferences from visual input provided in the form of images or videos. Typical tasks of computer vision include object recognition, scene understanding, image classification, visual tracking, etc. Feature representation is a crucial step for computer vision tasks. Image features are generally informative and discriminative in nature and can uniquely describe image contents [1,2,3]. The performance of visual tasks highly depends on the efficient representation of image features.

Before the advent of deep neural networks, handcrafted image features were constructed by extracting and grouping visual primitives that were manually defined according to human observation. They contain both top-down prior knowledge about specific tasks and intuitive visual understanding of how to manipulate abstractive image contents. Typically, domain experts manually select bottom-up discriminative visual entities (e.g., stimuli from color, shape, texture). With the development of deep neural networks, the image features can be extracted from deeply trained models without requiring explicit domain priors. The trained deep network model is able to directly extract the features from raw images that contain intrinsic and extrinsic properties that cannot be captured by handcrafted features. Recent work shows that the deep learned features significantly outperform the conventional handcrafted features in various tasks [4,5,6].

However, unlike handcrafted features with well-defined definitions of how to construct visual cues, deep features are still “mysterious” due to the complex processes of internal parameter updating, hierarchy feature learning, and non-linear feature map filtering. Despite providing remarkable performance, the ambiguous process of tuning millions of parameters on raw image pixels creates a barrier to understand how the features are learned and which steps are required to improve the performance. For example, the high computational cost is triggered by the need to train millions of parameters, some of which may not be necessary; this not only slows down the convergence, but also causes gradient vanishing and exploding, as well as overfitting. To alleviate these problems, methods such as dropout and a residual architecture are often applied. However, it is still difficult to tell whether the millions of parameters of the deep models play an equally important role or whether some of them are really unnecessary and even negatively affect the performance of vision tasks.

Therefore, the researchers wondered whether old-school handcrafted features still contribute to the new era of deep learning. If so, can we systematically incorporate the advantages of handcrafted features into deep networks? For example, Jin et al. [7] simply concatenated both handcrafted and deep features into a larger vector for recognition. Wu et al. [8] used additional fully connected layers on top of the original network to integrate both handcrafted and deep features for person re-identification tasks. These approaches, which simply combine both features without intervening in the internal deep learning process, lack practical solutions to the issues of deep networks. Therefore, their performance gains are limited.

**Figure 1:**
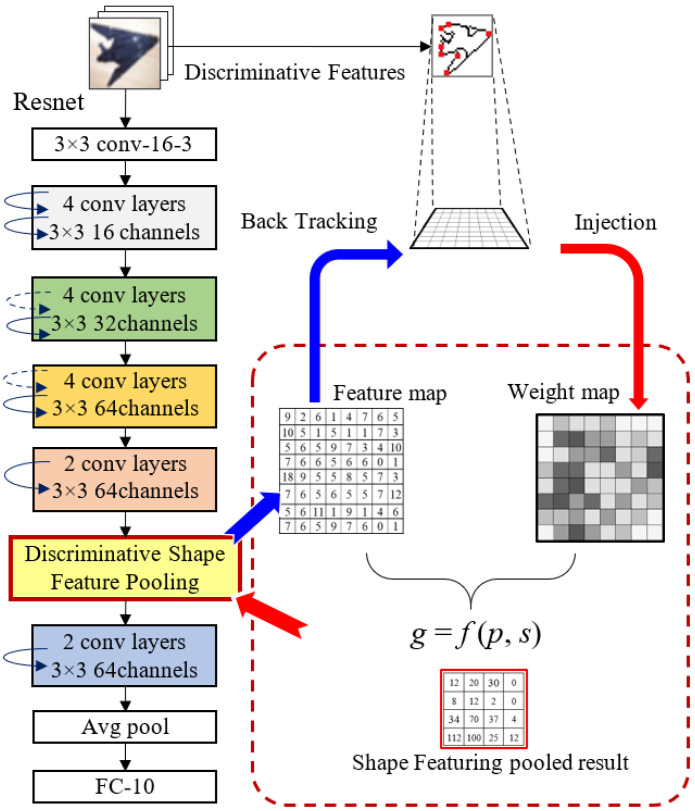
Architecture of DSF-Pooling in Resnet-18. Standard ResNet-18 only has the average pool right before the FC layer. Each convolutional block with curved arrows (skip connections) contains 6 convolutional layers. With the network hyperparameters, the feature map size is reduced from one block to another. The proposed DSF pooling layer is plugged into the networks (as shown in the yellow block).

In this study, we propose a novel Discriminative Shape Feature Pooling (DSF-Pooling) that injects the handcrafted discriminative shape features into deep networks to intervene in the parameter update process. The overall architecture of our approach is shown in Figure 1, where Resnet-18 was used as the CNN-based deep network.

Specifically, in the pooling stage, we adjust the original max pooled values according to the weights derived from external handcrafted shape-based perceptual features. The pooling mechanism of higher-level image features is similar to the visual feature selection processing in the human brain. The high-level pooling layers of the deep networks are modified by referring to the handcrafted features: Generic Edge Tokens (GETs) and Curve Partitioning Points (CPPs) [9]. The proposed approach has three main contributions as follows:

- Due to the intervention of the injected discriminative shape features, the model with DSF-Pooling obtains reliable performance improvement, especially for the natural scenes and living beings categories;
- Guided by the handcrafted features, the proposed deep network model reduces the learning curve, i.e., fast convergence;
- The proposed framework is generic and suitable for various handcrafted features and network architectures;
- The proposed pooling method has comparable performance to the other state-of-the-art methods.

## 2. Related Work

Feature representation is a crucial step for computer vision tasks. Researchers have been improving feature representation to gain better prediction results. The handcrafted features used for representation could be global features including shape (Hough transform [10], Zernike moments [11], etc.), color (color moments, color histogram, etc. [12]), and texture features (Gabor filters [13], random fields [14], etc.), which represent an entire image in a compact way. On the other hand, local image features such as SIFT [15], SURF [16], HOG [17], BRISK [18], etc., associated with different parts or regions of images provide more detailed image descriptions that are used on the Bag-of-Visual-Words (BoVW) technique [19] (inspired by text categorization) for constructing semantic image representations. The deep learning technique can automatically learn features from raw image data. The earlier-version Convolutional Neural Network (CNN), LeNet-5 [1], was used for recognizing digits, postal codes, etc., and then, the deeper Alexnet [20] brought revolutionary change in the field of computer vision. Jin et al. [7] simply concatenated the final feature representation from the trained Alexnet model with SIFT-based features encoded and pooled using the Local Linearity Coding (LLC) and Spatial Pyramid Matching (SPM) techniques. The prior knowledge of embedded handcrafted features and data distribution information from the CNN provided a feature representation that outperformed baseline methods for classification. Similarly, in [21], the color histogram was concatenated with the CNN feature vector for classification tasks. In the work of Wang et al. [22], color, texture, and morphology features were combined with CNN features to detect mitosis in medical imaging analysis. Those mentioned straightforward concatenation approaches only add additional data to the classifier, rather than providing a coherent way to improve the deep learning feature representation. Instead of simply concatenating handcrafted features with the final feature from deep networks, some CNN-based approaches utilize handcrafted features in a more comprehensive way. Kashif et al. [23] used processed color and texture features along with raw pixels as the input to a modified Spatially Constrained CNN (SC-CNN) [24]. Feature Fusion Net (FFN) [8] adds two additional layers on top of the seven-layer CNN, which fuses the color histogram and Gabor features with the last layer output of the Alexnet model. In this way, handcrafted features are combined into the later stage of the CNN to provide a supportive role for feature regularization. In DEFEATnet [25], each sequential layer has a SIFT feature extraction layer followed by sparse coding and local max pooling. The final representation of the network improved the performance compared with traditional methods, but the original deep learning techniques still performed much better. Wu et al. [8] used additional fully connected layers on top of the original network to integrate both features for person re-identification.

Looking into some typical deep networks, such as AlexNet [20], GoogLeNet [26], and ResNet [27,28,29], they are composed of repeating modules and structured by the branching and merging of various layers. Within each module, the pooling layer plays a vital role in achieving image transformation invariance, compact representation, yet effective expression in subsequent layers. Pooling essentially is a process of feature selection and stems from plausible biological evidence in high-level mammalian visual systems [30]. Spatial pooling has been a standard component of visual systems [31,32,33]. Besides the conventional max and average pooling approaches, stochastic [34] and S3pool [35] pooling randomly select a node in a neighborhood, while other approaches either use multiple [36] and adaptive pooling sizes [37] or take more data sources from multiple channels [38] and multiple scales to improve pooling performance. For example, He et al. [39] introduced SPP-net (CNNs + Spatial Pyramid Pooling layer), which accepts images of multiple input sizes and scales and produces a fixed-length representation of images. It showed improved results for classification and detection tasks. Gong et al. [40] performed orderless VLAD pooling on a CNN representation of image patches at multiple scales to obtain generic features. More recently, Saeedan et al. [41] proposed a weighted average pooling approach according to image details derived from handcrafted features. Local Importance Pooling (LIP) [42] utilizes learned weights as a subnetwork attention-based mechanism. SoftPool [43] downsamples the activation map based on the exponentially weighted sum of the original pixels within the kernel region (softmax normalization). This can improve the representation of high-contrast regions and present around object edges or specific feature activations.

Unlike others, our approach utilizes handcrafted features on each channel to influence the feature selection process. Specifically, it adjusts the original max pooled values according to the weights derived from external handcrafted features, shape-based perceptual features—Generic Edge Tokens (GETs) [9]. Thus, the domain knowledge can be utilized as the guidance to the final feature representation construction.

## 3. Proposed Method

Vision tasks often ask some high-level questions about an image, e.g., Does it contain a cat? Is it an elephant? Therefore, the system should be more sensitive to the entire input while being aware of key details. This goal can be accomplished by gradually aggregating information, eliminating irrelevant data, keeping important salience, and yielding coarser and conceptual representations. In a common deep neural network, the pooling layer stacked after the convolutional layer jointly carries out this task by choosing the dominant data and downsizing the feature maps. It not only reduces the computational complexity for upper layers, but also provides a form of spatial transformation invariance. Pooling is a process of feature selection and stems from plausible biological evidence in high-level mammalian visual systems. It plays a vital role in feature representation learning. As discussed in the related work, regular pooling approaches focus on the outputs from the previous convolutional layers lacking the ability of utilizing domain knowledge. We believe that by explicitly combing handcrafted features and convoluted feature maps, the pooling process could be better guided to produce enhanced outputs. Thus, we inject the external handcrafted image features into the pooling layer to guide the feature selection process during learning. The goal is to promote important data without losing details.

### 3.1. Handcrafted Discriminative Shape Features

**Figure 2:**
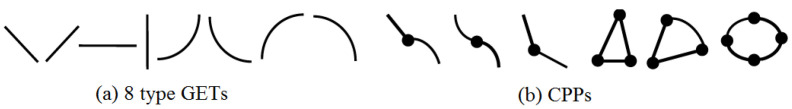
Handcrafted discriminative shape features. (**a**) lists 8 types of Generic Edge Tokens (GETs), along the edge curves; GETs are monotonic shape entities connected at Curve Partitioning Points (CPPs) listed in (**b**). GETs and CPPs are perceptual shape features that are extracted from edge contours and provide a set of basic shape descriptors for the semantic vocabulary.

Various types of handcrafted features are available for feature representation construction. Among them, the human visual perception highly relies on shapes. Contour-based features exploit object boundary information and are able to provide more detailed descriptions about object shapes. In our approach, we used the perceptual shape features: Generic Edge Tokens (GETs) and Curve Partitioning Points (CPPs) [9]. The GETs are considered as the shape primitives based on the principles of perceptual organization. They are categorized into eight different types. Four types depict the curve segments, and the other Four are straight line segments. Each CPP is a junction of two adjacent GETs, wherein the CPP indicates the shape saliency, i.e., the transition of perceptual shape geometry monotonicity. The combination of GETs and CPPs provides a set of mid-level shape descriptors as the visual vocabulary. Figure 2 shows the eight types of GETs and the corresponding CPPs. Although the perceptual GET and CPP features were selected in this work, other handcrafted discriminative features can also be utilized in our approach.

**Figure 3:**
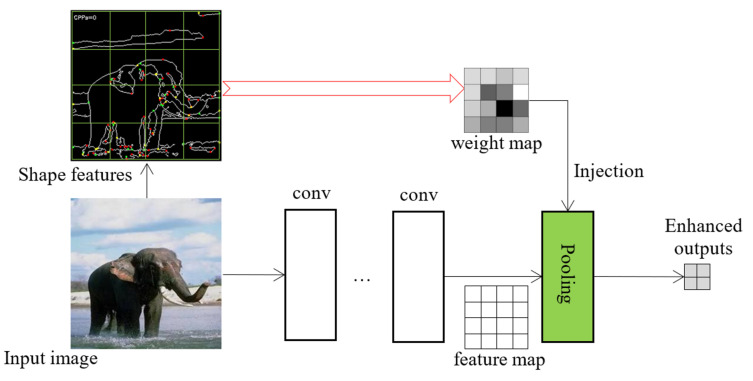
An example of feature weight map injection. The feature map from the convolutional layers is jointly processed with the injected weight map in the proposed pooling layer. The preprocessed handcrafted features are saved as files and are loaded into the network during training.

In order to measure the impact of these discriminative shape features, we define a weight $w_{i}$ for a local region *i* according to the feature occurrence in Equation (1).

(1) $$w_{i}=\frac{f_{i}}{\sum (f_{i})},0\leq i<r$$

where $f_{i}$ is the number of feature points in a local sub-region *i* and *r* is the count of sub-regions. The $w_{i}$ value is the ratio of GETs/CPPs within a local neighborhood and normalized between $\left[0,1\right]$. This ratio represents the local density of hand-crafted features and reflects the importance of a certain region. It will be used as the weight injected into the pooling layer of the deep network for producing the enhanced results. Figure 3 shows how the weight map is generated from an input image and injected into the pooling layer. The shape features are pre-extracted from the image; the size of the weight map is identical to the feature map from the convolutional layer. The cell intensities in the weight map represent the weight value of a local region computed by Equation (1), and the darker color means the density of the visual saliency is high in this local region, intuitively, where the vision attention should be drawn. The injected weight map guides the feature selection and reduction process. Prior to the deep learning era, there were many other classical handcrafted features, such as SIFT, SURF, and HOG. Their corresponding image descriptors have also demonstrated strong performance for various vision tasks. It is worth noting that our approach is open to any other features. In the ablation study, SIFT-based pooling was tested for comparison.

### 3.2. Discriminative Shape Feature Pooling

**Figure 4:**
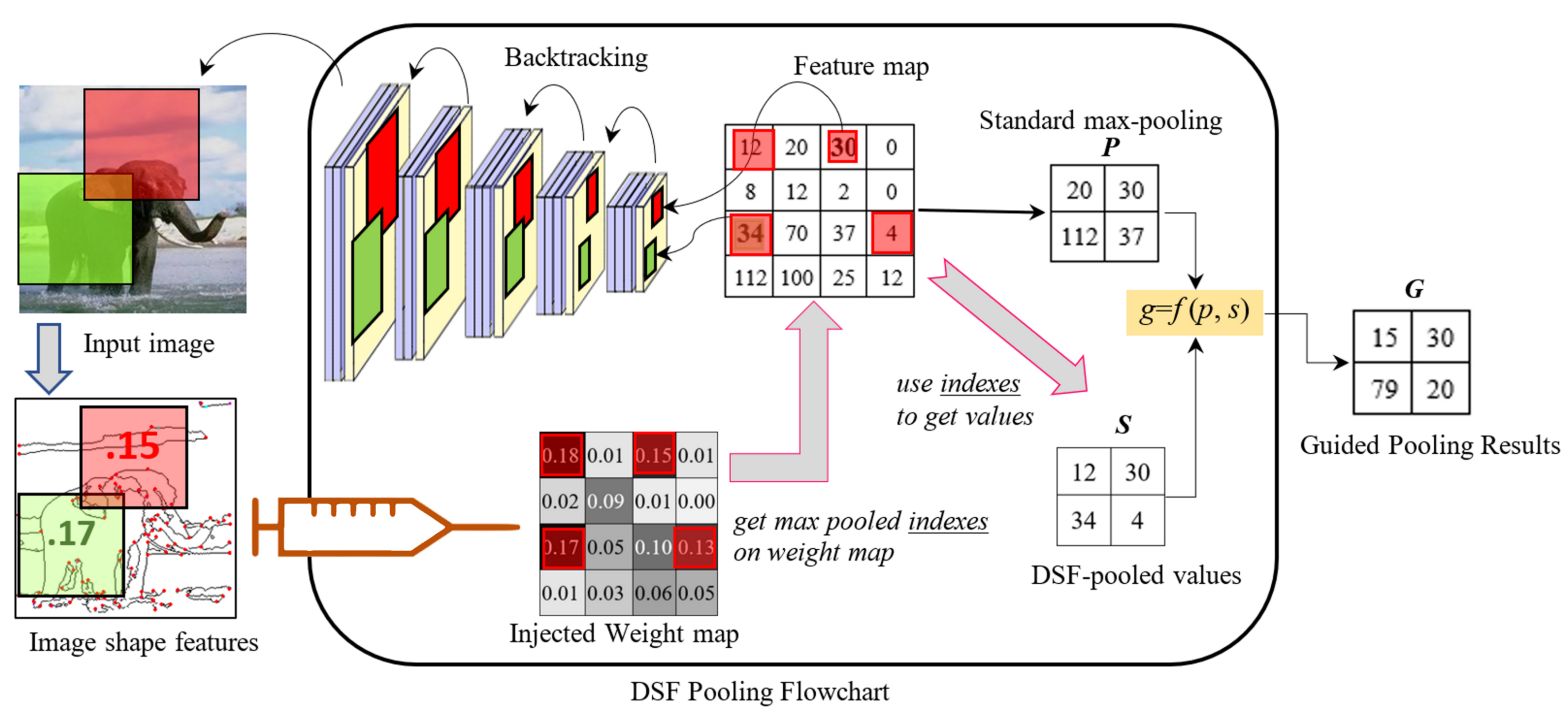
Flowchart of DSF-Pooling. Max pooled values P and the saliency S derived from the injected map are fused by Equation (2) to obtain the new pooled result G.

Pooling strategies play a crucial role in deep networks. An ideal pooling method is expected to extract only useful information and discard irrelevant details. Figure 4 shows the flowchart of the proposed pooling process. In the pooling layer, the regular max pooling method is still used to obtain the standard pooled value. The injected weights are utilized to adjust the max pooled values accordingly. Our goal is to boost the max pooled values if they are endorsed by the handcrafted features and suppress the max pooled values if they are disagreed by the injected weights.

Each element of the feature map has its receptive field on the original image, which can be backtracked through multiple convolutional layers. The visual importance of this receptive field is measured by the discriminative shape weight (shown in Equation (1)). The injected weight map collects the weights for all feature map elements and provides a different view of the convoluted data. In our approach, the pooling process decides whether to perform boost or suppression by checking both the feature map and weight map.

Specifically, max pooling is performed on both the feature map and injected weight map. We denote *p* as the standard max pooled value from the feature map. On the weight map, we track the indexes of the max values and use them to locate the values on the feature values, which is denoted as *s*. Both *s* and *p* are selected from the same spatial pooling region, but contain different aspects of the receptive field. *p* reflects the importance of the convoluted features; *s* carries handcrafted shape saliency where the domain knowledge is embedded. We boost the max pooled value if it is mutually agreed upon by both maps. If the maxed value is disagreed upon from the view of the weight map, we level it to the average value moderately. This principle is realized via a fusion function in Equation (2).

**Figure 5:**
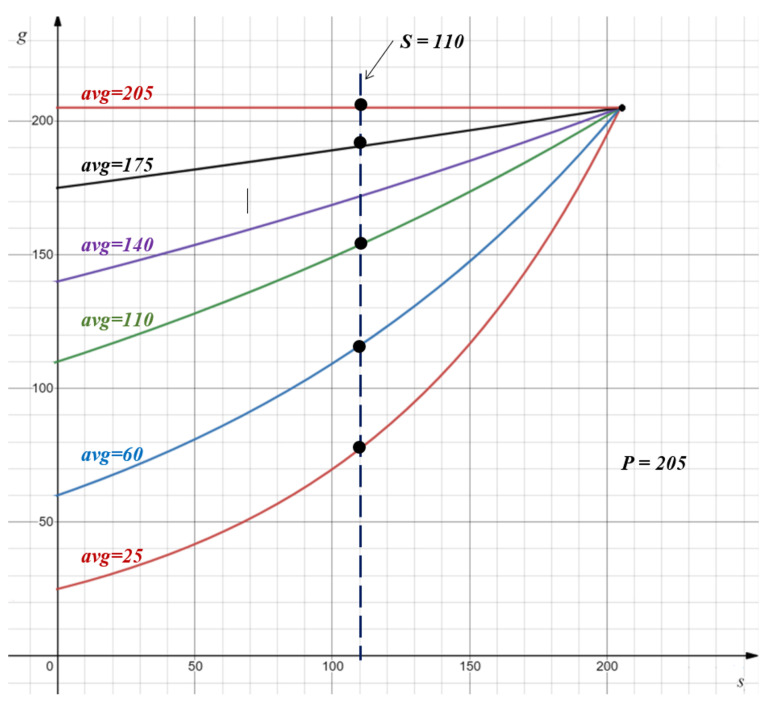
Data range of the pooled value. This plotted chart illustrates the relationship among *s*, *p*, and *g* in Equation (2). It lists 6 cases (curves) of average values (25, 60, 110, 140, 175, and 205). Assume that the regular max pooled value *p* is 205 and the max pooled on the weight map *s* is 110. The black dots are the output g of our pooling method, which are between the corresponding averages and the regular max one (205).

(2) g=f(p,s)=avg·exp(k·c)

where avg is the average value of the upsampling spatial region and $k=log(\frac{p}{avg})$ is the logarithmic ratio between *p* and avg. *k* indicates the significance of *p* in the sub-region, i.e., bigger *k* means *p* is more outstanding compared with its neighbors, and vice versa. *c* is related to the ratio between max pooled values *p* and *s*:

(3) $$c=\frac{\delta s}{\delta p}$$

where δs and δp are defined as s−min and p−min, respectively. Thus, *c* represents the level of consistency between the *s* and *p* values, ranging from 0 to 1. A larger *c* value means the selected salient feature is more consistent with the regular max pooled selection, while c=0 means both totally disagree with each other, i.e., s=min, p=max. Figure 5 visualizes the pooling function of Equation (2), where each curve represents the relationship between *g* and *s* given a max value (for example, p=205) and an avg value from a pooling region. The dots intersected with the vertical dashed line reveal *g* values for different avg when s=110. Note that each DSF-Pooling outcome *g* is ranged between the average and max values.

### 3.3. Case Study

As the example shown in Figure 4, handcrafted features GETs/CPPs are extracted from the image, and the weights are calculated for individual local sub-regions to form the weight map. The size of the weight map is identical to that of the feature map. By applying the backtracking operation, the local neighborhoods of the convoluted elements are found in the input image. Their pre-calculated weights can then be located in the injected weight map in the pooling layer. For example, the feature map elements 30 and 34 are mapped to the red and green sub-regions in the image, and their weights 0.15 and 0.17 are known to the pooling process. Then, max pooling is applied to both feature map and weight maps. *p* is the maxed values from the feature map. The maxed values from the weight map are not directly used. Instead, we take their position indexes (red squares on the weight map) to locate a set of different elements *s* from the feature map. *p* and *s* are fused to obtain *g*. Overall, the idea of the fusion function is that the pooling result *g* receives rewards if the regular max pooled value *p* is endorsed by the selection from the weight map (e.g., c≈1); otherwise, *g* would receive a penalty proportionally to the disagreement from both views (c<1), but it would not be less than avg. Compared with the results from DSF-Pooling and max pooling, DSF-Pooling values *g* present a higher contrast since the mutually agreed peak values from two views still keep the maximum, while others are suppressed due to their inconsistent opinions. Intuitively, DSF-Pooling provides boosted sharp outputs for promoting important data.

In sum, the new DSF-Pooling value is under the influence of the original pooling results (by reinforcing the final values to be between the average and maximum values of the original feature map). Meanwhile, it balances the visual semantic cues from the handcrafted features, which were totally ignored in the conventional pooling methods.

### 3.4. Algorithms

The integration of handcrafted shape features and the pooling process relies on two components: handcrafted feature extraction and backtracking and discriminative shape feature pooling value calculation.

Backtracking and Handcrafted Feature Extraction Preprocessing. Algorithm 1 shows the steps for backtracking from the feature map to the original image and corresponding shape features (GET/CPP).


**Algorithm 1:** Backtracking and GETCPP feature extraction.

**Input:** Input image I, preprocessed handcrafted features GETCPP()
    For each location x∈I,
      $M_{x}\leftarrow x⊙I,M_{x}$ is the mapped region
      $S_{GETCPP(M_{x})}\leftarrow \#GETCPP\left(M_{x}\right)$
      Compute w according to $S_{GETCPP(M_{x})}$ in this mapped region $M_{x}$ using Equation (1)
**Output:**GET−CPP feature-based weight map WM


Handcrafted features GETs and CPPs are pre-extracted from each image and are ready to be accessed during training and testing. The original input image I undergoes convolutional and pooling operations in the network to produce reduced-size feature maps based on the hyper-parameters of the network. Each location *x* on the feature map C is mapped to its corresponding region $M_{x}$ in I by backtracking. ⊙ is the mapping operation, which is based on the hyper-parameters of the network window size and stride for pooling layer and filter size, padding, and number of filters for the convolutional layer. Handcrafted features (GETs/CPPs) can be obtained according to $M_{x}$ to compute the weight using Equation (1). The output weight map is injected into the pooling layer.

Discriminative Shape Feature Pooling value calculation. Algorithm 2 describes the calculation of the DSF-Pooling values based on the convoluted feature map and the injected GET/CPP weight map.


**Algorithm 2:** Discriminative shape feature pooling.

**Input:** convoluted feature map CM, weight map WM
    For each pooling window in CM,WM
      $p_{i}\leftarrow valueOfMaxPooling\left(\mathrm{CM},w_{sz},w_{st}\right)$
      ${\mathrm{idx}}_{i}\leftarrow IndexOfMaxPooling\left(\mathrm{WM},w_{sz},w_{st}\right)$
      $s_{i}\leftarrow valueOfIndex\left(\mathrm{CM},{\mathrm{idx}}_{i}\right)$
      $g_{k}\leftarrow f\left(p,s\right)\left(Equation\left(2\right)\right)$
**Output:** New pooled values g∈G


Here, $w_{sz}$ is the pooling window size, $w_{st}$ is the pooling stride, and *k* is the index of pooled output *G*.

### 3.5. Back-Propagation

It is worth noting that all the steps inside the DSF-Pooling layer are differentiable, which means derivatives exist at all points. Due to the nature of the DSF-Pooling function (Equation (2)), the gradient ∇(g) with respect to the input $\left\{x_{i}\right\}$ in a pooling region has two special cases: g=avg and g=p, in each of which back-propagation computation is performed as the process for regular average or max pooling, respectively. Otherwise, ∇(g) is formulated in Equation (4) according to the chain rule:

(4) $$\frac{\partial g}{\partial x_{i}}=\left\{\begin{array}{c} \frac{g\cdot (1-c)}{sum}+g\cdot c\cdot \frac{p-min-p\cdot k}{p\cdot (p-min)},\;\mathrm{if}\;x_{i}=p\\ \frac{g\cdot (1-c)}{sum}+\frac{g\cdot k\cdot (s-p)}{{\left(p-min\right)}^{2}},\;\;\;\;\;\mathrm{if}\;x_{i}=min\\ \frac{g\cdot (1-c)}{sum}+\frac{g\cdot k}{p},\;\;\;\;\;\;\mathrm{if}\;x_{i}=s\\ \frac{g\cdot (1-c)}{sum},\;\;\;\;\;\;\;\;\mathrm{otherwise}. \end{array}\right.$$

where g,c,k,p,s,min are defined and explained in Equations (2) and (3) and sum is the summation of the pooling region. Additionally, look-up tables are used for storing and loading the max,min,s values during the forward and backward processes. The details of the formula proofs are shown in the Appendix A.

## 4. Experiments and Discussion

We first conducted ablation studies on the ResNet architecture. Then, DSF-Pooling was plugged into Alexnet on layer pool-5 to test on several benchmarks with pretrained models. Finally, to show the merits of this proposed approach, we compared it with state-of-the-art pooling methods for a classification task on ImageNet 1K.

### 4.1. Ablation Studies

ResNet-18 on CIFAR-10. We conducted experiments on CIFAR-10 using ResNet-18 [27] with 3 variants: Standard ResNet-18, ResNet-18 with max pooling, and ResNet-18 with DSF-Pooling. Standard ResNet-18 with only the average pool right before the FC layer was used as the baseline. There are three downscaling residual blocks in ResNet-18. For both max and DSF-Pooling variants, we modified the network by setting the stride in the third downscaling residual block to 1 and placing the corresponding pooling layer after this block. Hence, the shape of the output feature map of the modified network was consistent with the original ResNet-18. Figure 1 shows the architecture of our DSF-Pooling variant, where each block with curved arrows (skip connections) contains multiple convolutional layers. The standard nonlinear operations batch normalization and ReLU (not presented in the figure) were also applied after each convolutional layer.

CIFAR-10 consists of 50,000 color images for training and 10,000 images for testing. The original size of each image is 32×32 and enlarged to 40×40 by mirror padding. We trained all the models with Nesterov’s accelerated gradient with an initial learning rate of 0.1 and a batch size of 128. The training took 164 epochs with the learning rate reduced to 0.01 and 0.001 after around the 80th and 120th epochs, respectively. Our framework is open to any handcrafted features. To demonstrate this, we built a SIFT pooling by injecting the weights of classic SIFT keypoints into the pooling layer using Equation (2). This SIFT pooling layer was also evaluated in this study. As listed in Table 1, the experimental results showed both SIFT pooling and DSF-Pooling performed better than the max pooling variant and the baseline. DSF-Pooling achieved the lowest testing error at the final epoch, which indicates that the shape features provide stronger selection guidance than SIFT features.

ResNet variants on ImageNet 1K. ImageNet 1K [44] has 1.2 M images for training and 50 k for validation, distributed evenly across 1000 classes. We used 224 × 224 random crops (after rescaling) for training and testing on the validation set. The classification task on this dataset requires the methods to capture both global and discriminative details. We investigated whether the performance of the DSF pooling layer was consistent with deeper networks. We evaluated on 5 ResNet variants: ResNet-18, -34, -50, -101, and -152, respectively. Similar to the setting in the study of ResNet-18 on CIFAR-10, within each variant ResNet, we only placed a single DSF pooling layer immediately after the third downscaling residual block, while we set the stride in that residual block back to 1. During the training, we set an initial learning rate of 0.1 with an SGD optimizer and reduced it by a factor of 10 every 30 epochs for a total of 90 epochs. The number of epochs was chosen as no further improvements were observed for any of the models. We also set the batch size to 256 across all variant models. The result is summarized in Table 2. All results are reported on the validation set with single-crop testing. Adding DSP-Pooling lowered the error by 1.59%, 1.29%, 0.95%, 0.43%, and 0.61% for ResNet-18, -34, -50, -101, and -152 respectively. ResNet-101-DSP-Pooling was even lower than the standard ResNet-152 by 0.28%, despite having fewer layers and parameters. This demonstrates the effectiveness of our method across different network depths.

### 4.2. Alexnet with Pretrained Model

DFS-Pooling is a generic solution to a variety of deep networks. To demonstrate its generality, we applied it to Alexnet [20] with pre-trained models and tested it on several datasets. Alexnet consists of 5 convolutional layers followed by 3 fully connected layers, of which the first, second, and fifth convolutional layers are followed by max pooling layers for spatial sub-sampling. Among them, the output of the fifth layer represents high-level image features, containing major object parts. Hence, we plugged the DSF pooling layer into the fifth conv layer to examine the performance of the modified network. We used the model pretrained on ImageNet and trained and tested on the Caltech-256, Pascal VOC 2007 [45], Oxford-102 Flowers, and KTH Animals datasets, respectively.

Caltech-256. The Caltech-256 [46] dataset consists of 256 categories of images varying from man-made objects to natural scenery and living beings as well, along with an additional clutter category. The total number of images in the dataset is 30,607 with each class ranging from a minimum of 80 images to a maximum of about 827 images. The important aspect of the dataset is that the sizes of the images are different; although the categories are independent of each other, some of them are closely related. For our experiments, we selected 60 images per class for training, out of which 15 were considered for validation; the remaining images were used for testing. The results were evaluated based on the accuracy of the testing set. The classification accuracy of the baseline Alexnet model was 74.47%, while our method reached an accuracy of 76.13%. Table 3 shows the comparisons with several other approaches.

Besides the performance gain for the overall mAP, interestingly, the proposed method generally provided a larger performance margin for the categories of living beings on the Caltech-256, such as butterfly, gorilla, swan, etc. Table 4 shows the accuracy of some selected classes of the Caltech-256 dataset.

Pascal VOC 2007. The Pascal VOC 2007 [45] dataset has a significant variation of images in terms of size, orientation, illumination, position. and occlusion. It consists of 20 classes having a total of 9963 images split into 5011 training images and 4952 testing images. As the validation set, 2510 images of the training set were used. The classes of the dataset include birds; animals such as cat, dog, sheep, horse; vehicles such as aeroplane, bicycle, bus, motorbike, car, boat, train; indoor objects such as bottle, chair, sofa, potted plant, dining table, tv/monitor; and a class containing persons. The performance on Pascal VOC was measured by the mean average precision (mAP) over all 20 classes. The original Alexnet architecture provided a mAP of 80.21%, while our method performed better with a mAP of 81.45% (as shown in Table 5). A similar situation to the Caltech-256 dataset, our method performed better on the classes of animals and persons. For instance, the category of persons and birds showed a precision of 91.11% and 86.46%, respectively. Table 6 shows the breakdowns of 20 classes in the Pascal VOC 2007 dataset, which indicates that our approach outperformed the base model on some living beings classes.

**Figure 6:**
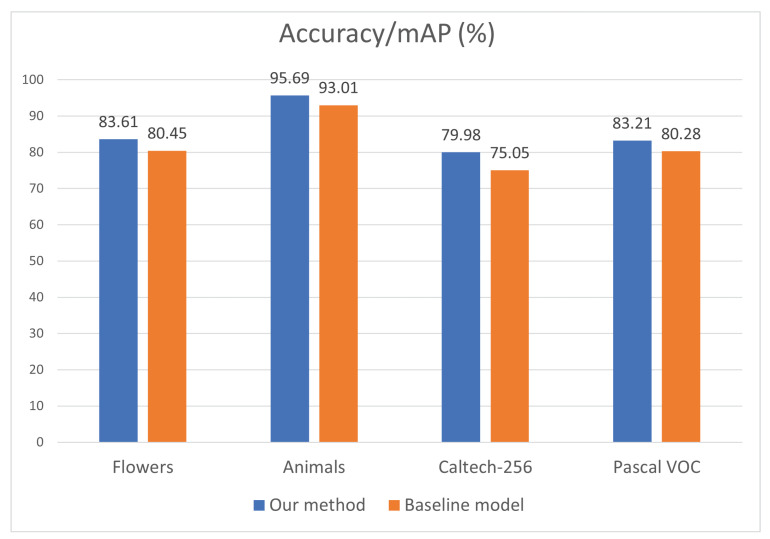
Result comparison on flowers, animals, and categories of living beings (animals, birds, insects, persons) from the Caltech-256 dataset and natural scenes (plants, flowers) from the Pascal VOC 2007 dataset.

Performance on living beings and natural scene objects. The result analysis on the Caltech-256 and Pascal VOC 2007 datasets showed that the classes containing living beings such as animals, birds, and persons, as well as classes in the category of nature performed considerably better compared to the baseline model. To further generalize the results, we chose the Oxford-102 flower dataset and the KTH animals [55] dataset to test our method. The Oxford flower dataset consists of 102 categories of different flowers, with each category having between 40 and 258 images. The total of 8189 images are split into training (2040), validation (1020), and testing (6149) sets. This dataset is challenging because there is great inter-class similarity and relatively lesser intra-class similarity. The KTH animals dataset has 19 classes with an average of about 80–85 images per class. Eighty percent of images from each class were used as training set, and the remaining twenty percent were used for testing. The performance on both types of objects is presented in Figure 6, where our method achieved 83.61% and 95.69% on flower and animal objects, which outperformed the baseline model by 3.16% and 2.68%, respectively. To reveal more details, a further study of the class-wise results on the Caltech-256 and Pascal VOC datasets was conducted. Specifically, the categories of living beings (animals, birds, insects, persons) from the Caltech-256 dataset and natural scenes (plants, flowers) from the Pascal VOC 2007 dataset were selected for testing. From Figure 6, our approach performed by 4.93% and almost 3% better on the selected Caltech-256 and Pascal VOC datasets. It can be concluded that our method achieved better performance for living beings and natural scene objects consistently.

### 4.3. Pooling Method Comparisons on ImageNet 1K

To further validate the effectiveness of our DSF-Pooling, we compared multiple pooling methods across several networks trained from scratch on the ImageNet 1K classification task. Among them, three DPP pooling layers were placed in the -ResNet-18, -34, -50, and -101 networks. Besides a max pooling layer, the LIP method puts 7 pooling layers in the networks. SoftPool only includes a single pooling operation after the first convolution block. The setting of S3Pool is similar to ours: put a single pooling layer after the third residual downscaling block. The comparison results are reported in Table 7. While outperforming stochastic and S3Pool methods with notable margins, the DSF-Pooling-based models performed similarly to other approaches. It is worth noting that, given the number of pooling layers used in the networks, the results highlight the merits of using DSF-Pooling.

### 4.4. Learning Efforts

**Figure 7:**
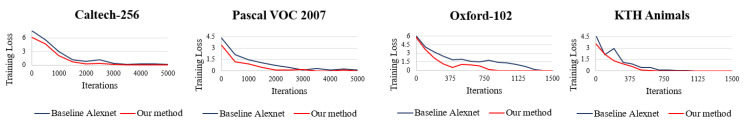
Learning curve (training loss vs. iterations) for the baseline Alexnet and the proposed method. Apparently, our approach converges faster during the training stage since domain knowledge embedded handcrafted features provide effective learning guidance.

By examining the learning curve for the baseline model and our method, we observed that our method converged more quickly. The guidance of handcrafted features helped the CNN model quickly reduce the loss, which indicates the efficiency of our method. The learning curves of all four datasets for Alexnet and our method are shown in Figure 7.

### 4.5. Visualization of the DSF-Pooling Results

**Figure 8:**
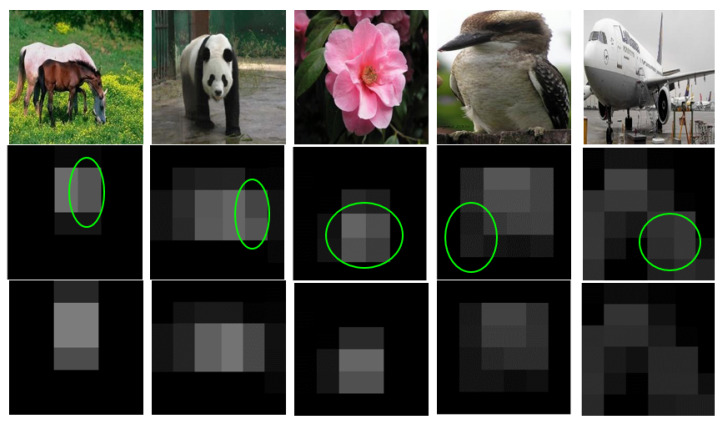
Visualization of learned feature maps from DSF-Pooling and max pooling. The second row and third row are from DSF-Pooling and max pooling layers, respectively. The DSF-Pooling layer reveals more semantics from image contents.

Figure 8 provides the visualization of two types of pooled layer-5 feature maps from the Alexnet. The first row shows the input images; the DSF-Pooling and max pooling results are presented in Rows 2 and 3 respectively. We can perceive that the DSF-Pooling scheme can discover and capture more details. For example, more background environmental contents were revealed in the horse, panda, and bird images; for the flower images, our output showed the different patterns for petal stamens; for the airplane images, our feature map can show the difference of the engine and wing parts. The visualization partially reveals the reasons for the performance difference.

## 5. Conclusions

The proposed novel DSF-Pooling method can guide the feature learning in deep neural networks with domain-specific prior knowledge. It extends the standard max pooling by utilizing the domain-specific prior knowledge in the following steps: (1) obtain the max pooling values from the feature maps of the convolutional layers; (2) backtrack the corresponding weights from the handcrafted shape features; (3) adjust the final values by weighting the two. The new results of the pooling layer were ranged between the average and max values and had a sharp distribution. The networks with the DSF pooling layer had a fast convergence speed and improved accuracy. The comparative experiments also verified that the deep learning model engaged with shape-based handcrafted features performed better for living beings categories. Additionally, our framework is generic and suitable for various handcrafted features and network architectures and achieved comparable performance to other state-of-the-art methods.

## Figures and Tables

**Table 1:** Experiments with ResNet-18 on CIFAR-10.

Model	Test Error (%)
ResNet-18	8.05
ResNet-18 Max pooling	9.12
ResNet-18 SIFT pooling	7.69
ResNet-18 DSF-Pooling	7.56

**Table 2:** Classification error rates (%) for ResNet variants on ImageNet 1K.

Network	Original	DSF-Pooling
ResNet-18	30.24	28.65
ResNet-34	26.71	25.42
ResNet-50	24.23	23.28
ResNet-101	22.31	21.88
ResNet-152	22.16	21.55

**Table 3:** Comparison of classification accuracy on Caltech-256.

Methods	Accuracy (%)
Sohn et al.’s convolutional RBMs, SIFT [47]	47.94
Huang et al.’s SIFT, improved Fisher kernel [48]	52.0
Bo et al.’s multipath HMP [49]	55.2
Zeiler and Fergus’ ZF-net [50]	74.2
Chatfield et al.’ 8-layer CNN [51]	77.6
Gao et al.’ DEFEATnet [25]	48.52
Baseline Alexnet model [20]	74.47
Proposed model	76.13

**Table 4:** Classification accuracy of Caltech-256 (selected classes).

	Butterfly	Cormorant	Elephant	Gorilla	Ostrich	Owl	Penguin	Hibiscus	Hawksbill	Christ	Swan
Alex	67.65	78.57	77.36	76.87	87.10	69.05	70.42	90.11	90.91	66.47	78.38
Ours	82.35	92.86	83.02	77.61	87.10	64.29	74.65	90.11	93.45	68.14	81.08

**Table 5:** Comparison of mAP on Pascal VOC 2007.

Methods	mAP (%)
Huang et al.—SIFT, improved Fisher kernel [48]	58.05
Sande et al.—SIFT, C-SIFT, OpponentSIFT, RGB-SIFT, rg-SIFT [52]	60.05
Razavian et al.—Overfeat [53]	77.2
Oquab et al.—transfer of mid-level CNN [54]	77.7
He et al.—SPP-net [39]	80.1
Chatfield et al.—8-layer CNN [51]	82.4
Alexnet	80.21
Ours	81.45

**Table 6:** Pascal VOC classification result breakdown.

	Aero	Bike	Bird	Boat	Bottle	Bus	Car	Cat	Chair	Cow	Table	Dog	Horse	Bike	PPL	Plant	Sheep	Sofa	Train	Tv
Alex	90.7	88.6	76.3	76.5	71.2	75.9	86.7	85.6	74.1	62.5	73.6	81.1	82.2	82.4	90.3	85.3	79.1	66.3	88.8	86.6
Ours	88.8	91.1	83.7	72.3	75.5	74.6	86.9	88.5	73.8	68.2	76.3	86.5	85.3	81.1	91.1	90.3	74.8	65.7	87.9	86.5

**Table 7:** Comparison of a variety of pooling methods on Imagenet1K for the classification task.

Method	Pooling No.	ResNet18	ResNet34	ResNet50	ResNet101	ResNet152
Original	-	69.76	73.3	76.15	77.37	78.31
Stochastic [34]	1	70.13	73.34	76.11	-	-
S3Pool [35]	1	70.15	73.56	76.24	-	-
DPP [41]	3	70.86	74.25	77.09	78.30	-
LIP [42]	7	70.83	73.95	77.13	79.33	-
SoftPool [43]	1	71.27	74.67	77.35	77.74	78.73
DSF Pool	1	71.35	74.58	76.72	78.12	78.45

## Data Availability

Not applicable.

## Appendix A

**Proof** **of** **Equation (4).** Suppose $X=(x_{1},x_{2},\cdots ,x_{m})$. Denote $avg=\frac{1}{m}\sum_{i=1}^{m}x_{i}$, $min=\min(x_{1},x_{2},\cdots ,x_{m})$, and $max=\max(x_{1},x_{2},\cdots ,x_{m})$. Let $s=x_{i}$, when i∈{1,2,⋯,m}, $k=\ln\left(\frac{max}{avg}\right)$, and $c=\frac{s-min}{max-min}$. Apparently, c∈[0,1]. Let $g=avg\cdot e^{k\cdot c}$. It is easy to see that g=avg when s=min and g=max when s=max. Otherwise, ave<g<max when min<s<max. Thus, for each 1≤i≤m, by the chain rule:

(A1) $$\begin{array}{ccc} \frac{\partial g}{\partial x_{i}} & = & \frac{\partial g}{\partial avg}\cdot \frac{\partial avg}{\partial x_{i}}+\frac{\partial g}{\partial s}\cdot \frac{\partial s}{\partial x_{i}}+\frac{\partial g}{\partial min}\cdot \frac{\partial min}{\partial x_{i}}+\frac{\partial g}{\partial max}\cdot \frac{\partial max}{\partial x_{i}}\\ & = & \left(\frac{g-g\cdot c}{avg}\right)\cdot \frac{\partial avg}{\partial x_{i}}+\frac{g\cdot k}{max}\cdot \frac{\partial s}{\partial x_{i}}+\frac{g\cdot k\cdot (s-max)}{{\left(max-min\right)}^{2}}\cdot \frac{\partial min}{\partial x_{i}}\\ & & +\left(\frac{g\cdot c}{max}-\frac{g\cdot c\cdot k}{max-min}\right)\cdot \frac{\partial max}{\partial x_{i}} \end{array}$$

Next, let us have a discussion about the partial derivative when *s* has different values. Case (i), If s=min, then c=0 and $g=avg=\frac{1}{m}\sum_{i=1}^{m}x_{i}$. Therefore,

$$\begin{array}{c} \frac{\partial g}{\partial x_{i}}=\frac{1}{m},\;\mathrm{for}\;\mathrm{each}\;1\leq i\leq m. \end{array}$$

Case (ii), If s=max, then c=1 and g=max. Therefore,

$$\begin{array}{c} \frac{\partial g}{\partial x_{i}}=\left\{\begin{array}{c} 1\;\mathrm{if}\;x_{i}=max,\\ 0\;\;\mathrm{otherwise}. \end{array}\right. \end{array}$$

Case (iii). If min<s<max, then $g=avg\cdot e^{k\cdot c}$. Since

$$\begin{array}{ccc} & & \frac{\partial max}{\partial x_{i}}=\left\{\begin{array}{c} 1\;\mathrm{if}\;x_{i}=max\\ 0\;\mathrm{otherwise} \end{array}\right.,\;\;\;\frac{\partial avg}{\partial x_{i}}=\frac{1}{m},\\ & & \frac{\partial min}{\partial x_{i}}=\left\{\begin{array}{c} 1\;\mathrm{if}\;x_{i}=min\\ 0\;\mathrm{otherwise} \end{array}\right.,\;\frac{\partial s}{\partial x_{i}}=\left\{\begin{array}{c} 1\;\mathrm{if}\;x_{i}=s\\ 0\;\mathrm{otherwise} \end{array}\right., \end{array}$$

using (A1), we obtain

$$\begin{array}{c} \frac{\partial g}{\partial x_{i}}=\left\{\begin{array}{c} \frac{g\cdot (1-c)}{sum}+g\cdot c\cdot \left(\frac{1}{max}-\frac{k}{max-min}\right),\;\mathrm{if}\;x_{i}=max,\\ \frac{g\cdot (1-c)}{sum}+\frac{g\cdot k\cdot (s-max)}{{\left(max-min\right)}^{2}},\;\mathrm{if}\;x_{i}=min,\\ \frac{g\cdot (1-c)}{sum}+\frac{g\cdot k}{max},\;\mathrm{if}\;x_{i}=s,\\ \frac{g\cdot (1-c)}{sum},\;\mathrm{otherwise},\; \end{array}\right. \end{array}$$

where $sum=avg\cdot m=\sum_{i=1}^{m}x_{i}$. That is,

$$\begin{array}{c} g\left(X\right)=\left\{\begin{array}{c} avg,\;\frac{\partial g}{\partial x_{i}}=\frac{1}{m},\;\mathrm{for}\;\mathrm{each}\;1\leq i\leq m,\\ max,\;\frac{\partial g}{\partial x_{i}}=\left\{\begin{array}{c} 1\;\mathrm{if}\;x_{i}=max\\ 0\;\;\mathrm{otherwise} \end{array}\right.,\\ avg\cdot e^{k\cdot c},\;\frac{\partial g}{\partial x_{i}}=\left\{\begin{array}{c} \frac{g\cdot (1-c)}{sum}+g\cdot c\cdot \left(\frac{1}{max}-\frac{k}{max-min}\right),\;\mathrm{if}\;x_{i}=max,\\ \frac{g\cdot (1-c)}{sum}+\frac{g\cdot k\cdot (s-max)}{{\left(max-min\right)}^{2}},\;\mathrm{if}\;x_{i}=min,\\ \frac{g\cdot (1-c)}{sum}+\frac{g\cdot k}{max},\;\mathrm{if}\;x_{i}=s,\\ \frac{g\cdot (1-c)}{sum},\;\mathrm{otherwise}\;. \end{array}\right. \end{array}\right. \end{array}$$

Note that in Equation (4), $p=\frac{\partial g}{\partial x_{i}}$, *p* is in lieu of max here. □
